# Do knowledge translation (KT) plans help to structure KT practices?

**DOI:** 10.1186/s12961-016-0118-z

**Published:** 2016-06-17

**Authors:** Salomon Tchameni Ngamo, Karine Souffez, Catherine Lord, Christian Dagenais

**Affiliations:** Institut National de Santé Publique du Québec, Montreal, Canada; Centre de Liaison sur l’Intervention et la Prévention Psychosociales (CLIPP), Montreal, Canada; Équipe de Recherche en Partenariat sur le Transfert des Connaissances (RENARD, FRQ-SC) [Knowledge Translation Research Partnership Team], Université de Montreal, Montreal, Canada

**Keywords:** Knowledge translation, Plans, Guidance, Scientific process

## Abstract

**Background:**

A knowledge translation (KT) planning template is a roadmap laying out the core elements to be considered when structuring the implementation of KT activities by researchers and practitioners. Since 2010, the Institut national de santé publique du Québec (INSPQ; Québec Public Health Institute) has provided tools and guidance to in-house project teams to help them develop KT plans. This study sought to identify the dimensions included in those plans and which ones were integrated and how. The results will be of interest to funding agencies and scientific organizations that provide frameworks for KT planning.

**Methods:**

The operationalization of KT planning dimensions was assessed in a mixed methods case study of 14 projects developed at the INSPQ between 2010 and 2013. All plans were assessed (rated) using an analytical tool developed for this study and data from interviews with the planning coordinators. The analytical tool and interview guide were based on eight core KT dimensions identified in the literature.

**Results:**

Analysis of the plans and interviews revealed that the dimensions best integrated into the KT plans were ‘analysis of the context (barriers and facilitators) and of users’ needs’, ‘knowledge to be translated’, ‘KT partners’, ‘KT strategies’ and, to a lesser extent, ‘overall KT approach’. The least well integrated dimensions were ‘knowledge about knowledge users’, ‘KT process evaluation’ and ‘resources’.

**Conclusions:**

While the planning coordinators asserted that a plan did not need to include all the dimensions to ensure its quality and success, nevertheless the dimensions that received less attention might have been better incorporated if they had been supported with more instruments related to those dimensions and sustained methodological guidance. Overall, KT planning templates appear to be an appreciated mechanism for supporting KT reflexive practices. Based on this study and our experience, we recommend using KT plans cautiously when assessing project efficacy and funding.

## Background

According to studies, few public health researchers and practitioners use an explicit framework when engaging in knowledge translation (KT) [1–4]. A KT planning template is a roadmap laying out the core elements to be considered when structuring the implementation of KT activities by researchers and practitioners. The various actors involved in KT processes usually operate intuitively, engaging in ad hoc activities according to their convictions and availability. Both the Institute for Work and Health and the Canadian Institutes of Health Research (CIHR) have invested considerable effort into providing guidance to researchers on KT practices and planning [5–8]. Growing numbers of funding agencies are also making KT plans a condition for funding [9]. A survey of 33 health research funding agencies that were proactive in promoting and supporting KT found that 73 % of them required KT plans [10]. Although few scientific studies have been conducted on KT plans, and fewer still on their impacts, the organizations and researchers that have implemented them report numerous benefits. Specifically, such plans can map out KT practices more clearly, ensure that KT is integrated into project management at an early stage, and foster the legitimacy and recognition of KT activities throughout the research process. They are also useful for taking users’ needs into account throughout the KT process, planning interactions with users, and formulating clear messages for specific audiences to optimize policy impacts and practice changes. There is widespread agreement in the scientific literature on the importance of planning KT because of the complexity of the process [7, 11].

The Institut national de santé publique du Québec (INSPQ; Québec Public Health Institute) is a major public health reference and expertise centre, with more than 600 employees. Its mandate includes producing and translating scientific knowledge and ensuring its dissemination in accessible language that supports decision-makers, practitioners and various partners in their initiatives to address the determinants of population health [12]. The INSPQ is part of a growing movement of organizations that see KT planning templates as useful tools for planning, leading, structuring and even evaluating KT. With this in mind, in 2010, it launched an organization-wide project to systematize its KT practices by means of KT plans. Twenty-two project teams were provided with sustained, customized guidance and reference tools to develop KT plans [13]. The guidance occurred at the team’s request. In the end, there were three to five customized guidance meetings provided along the KT process by the person in charge of KT advising at INSPQ. The team reflected on the key dimensions of their KT plan with counselling and a tool appropriation effort. The teams were made up of researchers and research assistants/coordinators and were asked to reflect on the most useful approaches to foster use of their knowledge (in terms of context, potential users, connections to be made with users, etc.) no matter at which stages they were in their project. The adviser had no control on the choices made by the team in the end since he acted as a guide. After more than 4 years of implementing and using KT plans, a study was undertaken to determine whether the resulting KT plans were found helpful by their users in improving the INSPQ KT processes.

The conceptual framework underpinning this study was drawn from various sources. It includes the key KT dimensions identified in the INSPQ’s reference document entitled *Animer un processus de transfert des connaissances* (Facilitating a Knowledge Translation Process) [13], which summarizes knowledge gleaned from 250 documents. This INSPQ document identifies seven KT dimensions: objectives of KT, content to be transferred, intended knowledge users, KT partners, KT strategies, analysis of KT barriers and facilitators (contextual analysis), and process stages (including KT evaluation). That document serves as a conceptual anchor for the KT consolidation and structuring process at the INSPQ. The INSPQ tool, entitled *Outil pour soutenir l*’*élaboration d*’*un plan de transfert de connaissances* (Knowledge Translation Planning Tool), is a KT planning template to support the development of a KT plan [14] that, while strongly inspired by that of Barwick [15], is directly derived from the abovementioned INSPQ reference document.

In this study, the various KT plan development processes were documented to determine what KT dimensions were used to structure the various teams’ KT approaches thus integrated into their KT plan. The dimensions to be considered were first identified through a review of the literature on KT plans and the INSPQ’s own reference guide, including a KT planning template [14].

It should be noted that this study was not intended to assess whether the KT plans were in conformity with the tools developed by the INSPQ, as the teams were expected to adapt those tools and use them flexibly, depending on what stage of development they had reached in their projects. The objectives of the study were (1) to identify known KT dimensions in the literature and then (2) to identify the dimensions that figured most often in the plans, highlighting those that appeared easiest or most difficult to operationalize. Identifying the dimensions integrated and its influential factors may help determine the appropriateness and applicability of actions planned around KT dimensions, which have yet to be empirically demonstrated [8, 14–17].

## Methods

The INSPQ KT planning was assessed through a mixed-methods case study of 14 projects. All 14 plans and associated documentations were assessed (rated) for dimensions integration using a contextualized analytical tool (see Analytical Grid in Appendix 1) and data from interviews with the KT project planning coordinators assessing the dimensions used and influential factors. The triangulation of multiple sources of information [18, 19] is chosen to ascertain that the information available in the KT plan of each project is corroborated in the diffusion of the process and results of the KT project and, with the interviews, to assess potential facilitators and barriers to KT planning at INSPQ.

### Development of the KT plan updated dimensions

Because the INSPQ’s reference document [14] was based on literature issued prior 2009, the more recent literature on KT plans was surveyed to enhance the framework and develop more generic methodological instruments that accurately reflect recent advances on the topic. A search was performed on the EBSCOhost platform and in the PubMed and ERIC databases using the keywords: knowledge t* (*translation*, *transfer*, *dissemination*, *utilization*, *application*) plan, planning, guidance.

Analysis of the scientific and grey literatures on KT planning corroborated the dimensions already in the INSPQ reference document and identified others [6, 10, 13, 15, 16, 20–22]. In all, eight dimensions were identified. The dimensions used in the current study are described in more details in the following sections. These refer to the KT process (e.g. analysis of context and of users’ needs, knowledge users, KT partners, KT strategies), guiding principles of KT (e.g. interaction with users, integrated KT approach) and conditions required to produce KT plans (e.g. leadership, resources, evaluation) (see KT dimensions identified most frequently in the literature in Appendix 1). Ultimately, the eight dimensions were used to analyse the KT plans (see analytical grid in Appendix 2) and to guide the interviews with the planning coordinators of the projects. Of note, the dimensions and their content were not classified nor presented here in a chronological order since KT is a process that requires several iterations along the evolution of the KT project, for example, KT evaluation may be planned at the beginning of the a KT project as a monitoring structure.

### Description of the eight dimensions

#### Analysis of the context (barriers/facilitators) and of users’ needs

Contextual analysis involves examining any barriers and facilitators that may present obstacles or opportunities for KT. These factors, whose numbers varied from one study to another, fell into three categories [14]: (1) factors linked to the knowledge to be translated, e.g. matching knowledge produced to users’ needs, clarity and accessibility of language, applicability of knowledge; (2) factors linked to actors, e.g. experience, credibility, interest in KT, openness, availability, motivation, attitude toward change; and (3) factors linked to organizational characteristics, e.g. availability of resources, support from managers, political climate, economic situation.

The analysis of users’ needs involves surveying the intended knowledge users at different times in the KT process: upstream from the project, to define the knowledge needs to be satisfied and to delineate the problem; during the project, to verify the users’ interest and receptiveness to new knowledge; after the knowledge has been produced, to identify any preferences regarding format and dissemination channels to be used; and after the knowledge products is developed, in order to pre-test it.

This dimension also encompasses the formulation of KT objectives based on the analysis of context and user needs.

#### Knowledge to be translated

This dimension refers to the three main types of knowledge that can be useful for public health action: (1) research-based knowledge (research and evaluation results); (2) tacit knowledge (intervention, management); and (3) knowledge derived from data analyses (administrative data and data on population health status and well-being). It also refers to the fit between knowledge and users’ needs and to the content adaptation required to make the knowledge clear, accessible and useful.

#### Knowledge users

This dimension refers to identifying, knowing about and setting priorities among potential users of the knowledge and other audiences, e.g. media, general public.

#### KT partners

The key actors who should be involved in the KT process need to be identified and their roles clarified. These are any individuals, groups, organizations or networks that might facilitate links with knowledge users. These actors may come from different sectors (academic, government, health and social services network, other areas of activity, the media, the general public) and may participate in various ways in the production, dissemination and use of knowledge.

#### KT strategies

Appropriate KT strategies need to be selected in accordance with the overall objective of the KT process and the type of knowledge to be translated, the knowledge users to be reached, the collaborations possible and the resources available. The KT strategies to be implemented for each knowledge user should be identified: determining the level of desired interaction, assessing the value of involving an intermediary depending on the strategy chosen, building on existing strategies, and identifying the best time to implement the strategy. Approaches that combine more than one KT strategy (multiple interventions) are recommended.

#### Overall KT approach

Broadly speaking, there are two main types of approaches to KT: integrated and end-of-grant. The integrated approach involves co-constructing knowledge with users from the outset and throughout the research process, whereas the end-of-grant approach calls for diffusion, dissemination or application of research results often in the early stage of discovery. Users and researchers may be involved in the development of targeted knowledge products or KT activities once the research process is completed.

#### KT evaluation

This dimension refers to evaluating the KT process and the impacts of the knowledge being translated in terms of its use and repercussions at the scientific, professional, organizational and socio-political levels.

#### Resources

This dimension has to do with different aspects related to the plan’s feasibility or to the conditions required for its development and implementation.

### Analytical grid and interviews

Each KT-related document collected was coded using a contextualized analysis grid (Appendix 1) developed based on the eight dimensions and 16 associated criteria. Each dimension’s level of integration was assessed, based on the criteria in the analytical grid, using a qualitative scale in which scores for each criterion were classified into three categories (predominantly, moderately, and hardly or not at all).

An interview guide was used to collect contextual information on the KT plans. It consisted of key questions divided into four groups: (1) general questions (e.g. At what point are you in your KT process? How did you go about developing your KT plan?); (2) questions on the process of developing KT plans (e.g. What are the strengths and weaknesses of your KT planning process?); (3) questions on the process of implementing the KT plan (e.g. Was your KT plan helpful to you in implementing your KT process? If yes, in what way? Did the KT strategies you used enable you to achieve your objectives and generate the desired outcomes?); and (4) questions on the conditions for carrying out the plan (e.g. what are the conditions required to develop and implement a KT plan? Do you think your project team members now have the competency required to develop and implement a KT plan on their own?).

### Sample

Of the 22 INSPQ projects available, 14 were included in the study because they were sufficiently advanced in the development of their KT plan at the time of testing (i.e. the plans selected for the study included at least four dimensions as well as contextual elements.) The teams were working in different areas of public health activity: competency development, population health analysis (e.g. monitoring social inequalities in health), infectious diseases prevention and control, the fight against sexually transmitted and blood-borne diseases, evaluation of healthcare services organization, workplace health, and healthy public policy, among others. The teams also varied in terms of their KT objectives – while some KT plans were geared toward making the team’s outputs more widely known, influential and useful through interactions with the intended knowledge users via appropriate channels (social media, discussion tables, government forums), others were more focused on co-constructing knowledge with knowledge users to foster adoption of a new health promotion or prevention approach, or on creating a process in which discussion, support and guidance helped facilitate the use of research results.

The selected sample was based on the significance of the cases in relation to the research topic, rather than a statistical model [23]. This was a non-probabilistic convenience sample. The 14 KT plans analysed and the 14 planning coordinators recruited for interviews were selected because they could provide information on the development of KT plans. The planning coordinators were in the majority researchers and research assistants/coordinators leading the team with no prior training in KT apart from the guidance delivered internally by the INSPQ. The team had previous experience in KT since they already were part of diffusion endeavours for research results, for example, and therefore had tacit knowledge about some KT practices but no formal training in KT, nor knowledge about KT science and its application.

### Data collection strategies and instruments

Initially, various documents were analysed such as the KT plans developed by each of the teams, minutes of meetings, reports of KT activities, scientific presentations (i.e. power point presentation, poster presentation, one pager, etc.) and unpublished working documents. Using the analytical grid, the KT plans and associated documentations were analysed from September to October 2013.

Interviews were then conducted with the team leaders. The 14 interviews were performed by one of the authors (STN, who did not participate in guiding the teams) from November to December 2013 and lasted, on average, 45 minutes. Only the planning coordinators of the project (or their representatives) were selected for interviews.

### Data analysis

The collected data underwent qualitative content analysis, in which the components of the plans were classified according to the KT dimensions and criteria developed [23–26]. STN, the principal investigator, performed the preliminary data analysis. The data were then compiled for each criterion and a mean was established to ascertain whether the dimension was ‘predominantly’, ‘moderately’ or ‘hardly or not at all’ integrated into the plans as mentioned in the analytical grid (Appendix 1). Means of the relative importance of each dimension were calculated as percentages, based on the criteria considered, to indicate the extent to which a dimension was integrated into the plan. To minimize subjectivity biases [27, 28], the coding exercise included an inter-rater agreement process leading to a consensus with the assistance of an independent researcher who had neither participated in the survey nor supported the team leaders. The data analysis was validated by the project team coordinators, who were thoroughly familiar with the plans. When analysed inductively, the data revealed convergences/divergences among several plans and/or the specific traits of each plan and the KT dimensions.

All interviews with the KT planning coordinators were recorded and transcribed. The interviews were analysed in six steps [27]: (1) transcription of the interviews; (2) reading of the transcripts and data coding; (3) categorization or classification of relevant information based on the KT dimensions identified; (4) quantification of responses to compile data; (5) cross-sectional analysis of the data from each project; and (6) interpretation of the results and insertion of interview extracts into the articles. Significant situations complemented the results of the documentary analysis.

## Results

The results presented in this section are based on analysis of (1) the documents collected from the 14 projects and (2) transcripts of the interviews with planning coordinators. Table 1 presents an overview of all the results and the following sections addresses the results for each dimensions analysed combining the results from the documentations analyses and from the interviews. Altogether, five dimensions appeared to be more integrated: ‘analysis of the context and of users’ needs’, ‘knowledge to be translated’, ‘KT partners’, ‘KT strategies’ and, to a lesser extent, ‘overall KT approach’. On the other hand, three dimensions appeared to be less well integrated: ‘knowledge about the knowledge users’, ‘KT evaluation’ and ‘resources’.

**Table 1:** Rating of knowledge transfer (KT) dimensions integration into KT plans

KT Dimensions	Criteria	Rating of dimensions integration (%)		
		Predominantly	Moderately	Hardly or not at all
Analysis of context (barriers/facilitators) and of users*’* needs	Identification of the problem or of the need for knowledge (identified objectively or intuitively)	64	36	0
	General KT objective defined	100	0	0
	Specific objectives defined	71.5	21.5	7
	KT context: analysis of opportunities and obstacles	57	14	29
	Mean	73	18	9
Knowledge to be translated	Types of knowledge (several vs. one main type)	71.5	14.5	14
	Suitability to the needs of knowledge users	57	36	7
	Adaptation of the contents (actions and intentions)	86	14	0
	Mean	71.5	21.5	7
Knowledge users	Identification and prioritization of knowledge users	64	36	0
	Knowledge about the knowledge users’ characteristics and preferences	14	57	29
	Mean	39	46.5	14.5
KT partners	Key actors to be involved (individuals, organizations, groups and networks identified, roles defined)	71	29	0
KT strategies	Choice of KT strategies to be implemented in line with objectives	86	14	0
	Multiple interventions, including dissemination and uptake strategies	100	0	0
	Implementation of strategies with detailed steps and follow-up mechanisms	36	21	43
	Mean	74	11.5	14.5
Overall KT approach	Integrated approach (co-construction of knowledge from the outset and throughout the research process)	43	50	7
	End-of-grant approach (user and/or researcher involvements to guide development of targeted knowledge products or KT activities, and tools	43	50	7
	Mean	43	50	7
KT evaluation	Evaluation of the KT process planned and methods defined	7	43	50
Resources	Feasibility with regard to availability of human, material and financial resources	36	0	64

### Analysis of the context (barriers/facilitators) and of users’ needs

The ‘analysis of the context and of users’ needs’ dimension appeared well integrated in most of the plans. This was more apparent in the criteria related to identifying KT objectives (general and specific) and knowledge needs than in the criterion regarding context analysis. Even though present in the majority of the plans, context analysis was found to be the least well integrated criterion for this dimension (43 % of the plans had included it ‘moderately’ or ‘hardly or not at all’). Based on the content analysis of the interviews with the planning coordinators, this seemed more to reflect the difficulty of analysing the context than the importance attributed to it by the planning coordinators. Several excerpts from the interviews reflect this: “*… dimensions like the analysis of context … are harder to achieve*”, “*… analysis of context is very important and takes a lot of time, it should not be given the same weight as is given to the other dimensions*”, and “*… analysis of context is not easy to define*”. From the interviews, it is clear that, despite the difficulty of integrating it into the plans, respondents perceived it as one of the most important and structuring dimensions for the KT process.

### Knowledge to be translated

Most of the plans considered different types of knowledge to be translated, such as research-based knowledge, tacit knowledge and knowledge obtained through data analysis. The fact that the KT plans took into account tacit knowledge did not, however, mean they all included co-construction of knowledge and participatory research upstream from the project. For example, data on the ‘overall KT approach’ dimension revealed that half of the planning processes began only after the knowledge had been produced, which means tacit knowledge was recognized more often in the KT strategies planning stage than in the research stage. Even though, with regard to content, efforts were made to incorporate a variety of types of knowledge that could be useful for action, it appeared that the knowledge products specified in the plans were not always appropriately matched to the needs of knowledge users, with 57 % reporting predominantly being suitable for the needs of knowledge users. From the interviews, several possible explanations were raised: there were many potential users, such that lack of time and resources made it impossible to satisfy all their various needs; their needs may not have been clearly defined; and the knowledge products were predetermined at the outset of the mandate (without any users being consulted about their needs), leaving little leeway to develop products for other potential users. Of note, the vast majority of the plans (86 %) included measures for putting content into a user-friendly format, in language that would be accessible to the intended knowledge users.

### Knowledge users

Identifying knowledge users and setting priorities among them did not appear to pose a problem, as 64 % of the teams had carried out that exercise. However, only 14 % of the plans described the characteristics and preferences of the knowledge users in detail. As one respondent remarked: “*The reflex for getting to know the knowledge users is missing*”. This may seem to contradict the data indicating that nearly half of the plans called for regular interactions with users (see the ‘overall KT approach’ dimension), which suggests better knowledge about the knowledge users. However, given the limited number of users with whom it is possible to maintain ties during the KT process, it may be that the sample was too small to fully capture the characteristics of the group it represented. From the interviews and the integration ratings, it appears that the main challenge related to knowledge users has to do with getting to know them.

The knowledge users were diverse and varied from one team to another. The groups most often addressed in the plans were (1) professionals and decision-makers from various ministries and partners from different government sectors; (2) decision-makers, managers and practitioners in public health at the regional and local levels; (3) associations, non-profit organizations and private foundations; (4) researchers in universities and academic networks; (5) health professionals (physicians, microbiologists, infectious diseases specialists, psychiatrists, nurses, other clinicians) from various healthcare organizations; and (6) directors, teachers and professionals working in complementary school services.

### KT partners

All the plans identified the partners, whether individuals or groups, who could be helpful in reaching knowledge users, with 71 % rated as ‘predominantly’. The interviews confirmed this observation, with about the same proportion (i.e. 8/14) of the planning coordinators indicating that they did not have to assert any particular leadership to mobilize the individuals concerned by their project. Moreover, they reported that, in settings that were either not very supportive or less receptive to the KT process (i.e. 6/14), local facilitators and information brokers were mobilized as needed to overcome that reluctance.

### KT strategies

With regard to the ‘KT strategies’ dimension, we noted that all the plans (100 %) called for multiple interventions, as recommended in the literature. Moreover, 86 % of the plans were based on KT strategies that were in keeping with the objectives. Even though most of the teams were in the implementation phase of their plan at the time of the study, it appeared that there was no detail step and follow-up mechanism about the strategies used during implementation in 43 % of the plans. This poor rollout of KT strategies may be due to a lack of experience or practice, the teams’ inadequate familiarity with the dimensions of a KT plan, a lack of support (many teams’ went autonomous at that stage) or insufficient resources. Regardless of the type of project, based on the content analysis of the plans, three types of KT strategies appeared to be emphasized in most of the plans: (1) dissemination of the product on a website or information literacy portal; (2) oral or poster presentations (conferences, symposia, conventions, etc.); and (3) discussion and consultation activities with different groups and working committees.

### Overall KT approach

Half (50 %) of the KT planning processes began only after knowledge production had been completed. A number of coordinators pointed to a lack of KT planning at the beginning of their project: “*The plan should have begun a bit earlier…*”, “*priority users were consulted too late, after the knowledge had been produced*”. In this respect, the KT planning processes appeared to have raised the teams’ awareness of the need to incorporate KT as early as possible into their project.

### KT evaluation

In half of the plans (50 %), the ‘evaluation’ dimension was almost non-existent. Most often, no evaluation had yet been planned, even though in some of the interviews the desire to produce certain outcomes, such as adopting a policy, changing practices or implementing a guide, was discernible. In some interviews it was noted that satisfaction was informally acquired but that no formal KT evaluation had been done yet.

### Resources

There was scarcely any indication of the plans’ feasibility, as 64 % made little or no mention of resources to be mobilized. Based on the planning coordinators answers in the interview, we argue that this may be because the teams had difficulty estimating the resources needed to carry out their plan, as they had little information about and experience with operationalizing KT strategies (phases, costs, etc.). It may also be that this dimension was addressed more explicitly in the project mandate or specifications. As one respondent explained: “*The skills are very broad and require resources and expertise that can help us stay on course and move forward*”. Another added: “*Time and resources are not always available*”, while a third noted that “*Each project is different and specific, and despite the training we were given, we are not experts in KT*”.

In addition to tools and resources, the teams expressed serious needs for guidance, both to help them take into account the most problematic dimensions of a KT plan and to support the development process overall. Several planning coordinators felt that occasional consultations with KT specialists on specific topics would be sufficient to help them make effective progress and be more autonomous in developing a KT plan if required in the future. However, roughly 50% of the respondents said they would not have the knowledge, skills or expertise to develop a KT plan in another context without sustained guidance, nor would they be able to find the time to do it without being guided properly by a KT specialist. In all cases, there would be a need for periodic or ongoing support.

## Discussion

### Main findings

Several authors have recommended conceptual frameworks to guide and plan the KT process so that initiatives are coordinated, linked and formalized [6], with some also having developed guides to structure KT plans. The aim of this study was to determine whether and how the dimensions in these frameworks were taken into account by the scientific teams and which dimensions were considered. The research question was prompted, in part, by the fact that several planned action theories are structured around dimensions that should be considered in guiding the desired change, but without those dimensions’ applicability or relevance having been empirically verified [2, 29–31].

This study showed that none of the dimensions was perfectly integrated into all the plans. This was probably due to the wide variety of mandates, projects and contexts within which the teams were working. The study’s participants did not believe it was always possible to fully incorporate all the dimensions. For example, at the start of a project, it is sometimes difficult to determine the intermediaries involved, as the knowledge users are not yet well defined. The dimensions are easier or more difficult to plan depending on where the project is in its evolution. These results show how flexible or inflexible KT plans might be when it comes to integrating a dimension, corroborating other studies that have shown that using a guide or a framework to orient KT is useful to the extent that there is flexibility in its application [7, 8, 11].

Nonetheless, some dimensions appeared more often than others in the KT plans, despite the varied nature and contexts of the projects. The dimensions ‘analysis of the context and of users’ needs’, ‘knowledge to be translated’, ‘KT partners’, ‘KT strategies’ and, to a lesser extent, ‘overall KT approach’, were strongly integrated into more than 70% of the plans. While this remains to be confirmed by other research, it is therefore to be expected that these dimensions would be more easily incorporated into KT plans, even if some of them, e.g. analysis of the context and of users’ needs, are more challenging to implement. With this in mind, funding agencies that require an analysis of the context and of users’ needs in a KT plan evaluation should weight this dimension according to the difficulties researchers experience when trying to operationalize it. Tools, techniques and guidance with proven validity or usefulness could also be provided to better guide and support them.

Three key dimensions – ‘knowledge users’, ‘KT evaluation’ and ‘resources’ – seemed more difficult to integrate into the plans. These dimensions posed a challenge for several teams, who did not feel sufficiently equipped to account for them in their plans. Some individuals (4/14) found that the mechanisms (process, method, etc.) and terminology (vocabulary) were abstract and had to be learned. Indeed, certain authors have noted this difficulty and have concluded that KT planning requires a period of adaptation and learning, much like the training modules developed to equip researchers and reviewers to understand the different KT dimensions [7, 8, 11, 14]. The results of the present study are in line with these findings, in particular, for the three abovementioned dimensions, which needed more explanation and operationalization. In fact, very few practical resources (tools, guides) are available to help researchers identify the characteristics and preferences of their knowledge users, estimate the costs and resources required to carry out KT activities, or evaluate a KT process. Given that all the dimensions are important, a solution to improve those that appear more difficult to integrate is to provide professional KT support to researchers and practitioners, who, for the most part, do not have the competencies to carry out operationalizable KT plans. Of course, KT tools, such as a KT planning template and training, help researchers and practitioners in developing their KT plan. For some dimensions of the plan, as those mentioned and our observations, researchers and practitioners require appropriate professional KT support.

As for KT evaluation, even though this dimension is of utmost importance to funding agencies and scientific organizations engaged in KT, it appears to be the most challenging part of KT planning. More research is needed to help disentangle KT process and impact evaluation as well as to help provide indications for best practices in KT evaluation. Many questions remain with regards to who should support such endeavours financially and professionally; for example, should part of the KT budget allocated by funding agencies be systematically dedicated to KT evaluation and should the responsibility for evaluation be assigned to KT specialists, to ensure adequate time and attention are allocated to it?

Advocates of contribution analysis in impact assessment stress the importance of using a planned, structured approach to demonstrate how the KT strategies were carried out and the desired outcomes achieved [30]. In this view, KT plans are a useful means for measuring the gap between what was planned at the outset and what was actually achieved. However, to evaluate the outcomes of their KT process, scientific teams need practical and specific guidelines that concretely explain what to measure, when, and for how long, depending on context, and that take into account particular aspects of KT evaluation (such as the challenges of causal attribution). Organizations also need to explicitly describe their vision of KT to provide teams with a common foundation for evaluation.

In short, we can conclude that KT plans are useful for clarifying KT-related choices and developing reflexive practice, but that they need to be combined with other resources and support. Goering et al*.* make the same observation when they note that: “*Plans are necessarily more limited when settings do not have adequate resources and support in terms of communication specialists and infrastructure to assist researchers in preparing proposals*” ([3], p. 99). Also identified in the interviews as essential conditions for successful KT plan implementation were support from the organization, the commitment and involvement of everyone involved, and particularly administrators, managers and political leaders, and the assignment of a specific mandate linked to KT.

### Limitations

One of the key limitations of the study concerns the subjectivity (partiality) inherent in the evaluation process [27, 28]. Aside from evaluator bias, certain criteria, such as the fit between knowledge produced and knowledge users’ needs, are open to different interpretations. In this regard, an inter-rater agreement exercise involving an expert who was not among those guiding the project teams in developing KT plans was helpful in detecting disparities in the use of the analytical grid and testing the quality of the evaluation process. A third person was involved as needed to resolve any cases of disagreement. Another limitation has to do with case study method and the difficulty of generalizing observations based on a single field. As such, the study’s findings are presented as avenues for reflection and require further investigation. Finally, another limitation of this study lies in the fact that it describes the plans at a given point in time. Between the time of data collection and the present moment, the plans have most certainly evolved and some dimensions may since have been completed and even operationalized.

### What this study adds: implications for practice

Several research projects have paved the way for this study [7, 10, 11, 13, 15] by developing precise guidelines for the development of KT plans. In our study, we sought to take these studies further by empirically verifying whether such guidelines are actually helpful for structuring KT plans. In this regard, the study represented a step forward in testing the practical application of KT plans. It identified the dimensions around which a plan should realistically be evaluated. While earlier studies have shown that KT plans are limited instruments that require tools and additional support, our study goes further by specifying which dimensions are the most complex to grasp. In short, our study contributes to the development of a body of knowledge on KT planning practices that is still in its early days.

## Conclusion

This study outlines the strengths and weaknesses that could potentially put into perspective whatever impact the plans will or will not manage to produce. However, it does not inform on how the presence or absence of certain dimensions in KT plans will influence those plans’ ultimate effectiveness.

Meanwhile, this study showed that the plans were useful for clarifying KT-related choices and could also serve as project management tools for the teams. Absent proper support, the KT plans’ structuring effect was seen mainly in the reflexive approach they engendered rather than in any systematization of practices.

It is clear that KT planning templates, in themselves, are of limited use for guiding project leaders in their KT process. Funding agencies and scientific organizations that embark upon this path need to implement additional support mechanisms such as periodic consultation and ongoing guidance, methodological development and training. Moreover, they need to use KT plans cautiously when assessing project performance and funding. The results of this study showed that the integration of core KT dimensions was far from being systematized or generalized. Several dimensions were not integrated simply due to the lack of appropriate resources and competencies.

## Abbreviations

INSPQ, Institut national de santé publique du Québec; KT, Knowledge translation

## Appendix 1

**ᅟ:** KT dimensions identified most frequently in the literature

KT dimensions	Number of authors and references
1. Analysis of context (barriers/facilitators) and of users’ needs	18 [1–4], [8, 9], [11], [14, 15], [17–24]
2. Knowledge to be translated	15 [1, 2], [4], [8, 9], [11], [14–19], [22], [23, 24]
3. Knowledge users	15 [1–3], [8, 9], [14–23]
4. KT partners	15 [1–3], [8, 9], [14–19], [21], [24]
5. KT strategies	17 [1–4], [8, 9], [11], [14–22], [23]
6. Overall KT approach	15 [1–3], [8, 9], [14–16], [18–22], [23, 24]
7. KT evaluation	10 [1, 2], [8, 9], [14–19]
8. Resources	18 [1–4], [8, 9], [11], [14–19], [21–24]

## Appendix 2

1. Information on the project teams

Project title:

Team coordinator:

Scientific unit:

2. Analysis and evaluation

**ᅟ:** KT plan and analytical grid

KT plan development: analytical grid			
Dimension 1: Analysis of the context and of users’ needs			
Criterion	A (Predominantly)	B (Moderately)	C (Hardly or not at all)
Identification of the problem or the need for knowledge	The problem or the need for knowledge that led to the KT process was verified among knowledge users	The problem or the need for knowledge was identified intuitively	The problem or the need for knowledge was not identified
KT Objectives	The general KT objective is defined from the viewpoint of the mandate/project that it is intended to support	The general KT objective is defined but not linked to the mandate/project that it is intended to support	The general KT objective is not defined or specified
	Specific objectives are defined for each of the knowledge users	Specific objectives are defined for a few knowledge users	Specific objectives are not defined based on knowledge users or are not specified
KT Context	KT opportunities and obstacles were analysed and mechanisms/solutions were identified	KT opportunities and obstacles were analysed but the corresponding mechanisms/solutions have still not been or were not identified	The plan does not include an analysis of the KT context
Dimension 2: Knowledge to be translated			
Criterion	A (Predominantly)	B (Moderately)	C (Hardly or not at all)
Types of knowledge	The KT process is based on the three main types of knowledge: research-based knowledge, tacit knowledge, and knowledge derived from data analysis	The KT process is based on two of the three main types of knowledge	The KT process is based on one main type of knowledge
Fit with knowledge users’ needs	The knowledge to be produced or translated fully satisfies the users’ need(s) for knowledge	The knowledge to be produced or translated partially satisfies the users’ need(s) for knowledge	The knowledge to be produced or translated does not satisfy the users’ need(s) for knowledge, or may do so but the needs are not explicitly identified in the plan
Content adaptation	Measures are planned to make the content clear, accessible and useful to the knowledge users	There is an intention to make the content clear, accessible and useful to the knowledge users, but no measures are planned	No effort has been made and there is no intention in the plan to make the content clear, accessible and useful to the knowledge users
Dimension 3: Knowledge users			
Criterion	A (Predominantly)	B (Moderately)	C (Hardly or not at all)
Identification and prioritization of knowledge users	The different knowledge users to be reached have been identified and classified by priority	The different knowledge users to be reached have been identified but have not been classified by priority	The different knowledge users to be reached have not been identified
Knowledge about the knowledge users	The preferences and characteristics of the knowledge users have been described in detail	The preferences and characteristics of the knowledge users have been identified in a general way	The preferences and characteristics of the knowledge users have not been identified
Dimension 4: KT partners			
Criterion	A (Predominantly)	B (Moderately)	C (Hardly or not at all)
Key actors (individuals, groups, organizations and networks) to be involved	All actors concerned by the KT process, (partners, intermediaries, potential opponents, etc.) have been identified and their roles defined	The actors concerned by the process have been identified but their roles have not been defined	The actors concerned by the process have not been identified
Dimension 5: KT strategies			
Criterion	A (Predominantly)	B (Moderately)	C (Hardly or not at all)
Choice of KT strategies to be implemented	The strategies selected are consistent with the objectives identified	Most of the strategies selected are consistent with the objectives identified	The strategies selected are hardly or not at all consistent with the objectives identified
Multiple interventions	The plan is based on multiple interventions that combine dissemination and uptake strategies	The plan is based on multiple interventions that focus mainly on a single type of strategy (dissemination or uptake)	The plan is not based on multiple interventions
Implementation of the strategies	The implementation stages for all the KT strategies are presented in detail and monitoring mechanisms are planned to ensure they are carried out	The implementation stages for at least one KT strategy are presented in detail and monitoring mechanisms are planned to ensure it is carried out	The implementation stages for the KT strategies and monitoring mechanisms are not presented in the plan
Dimension 6: Overall KT approach			
Criterion	A (Predominantly)	B (Moderately)	C (Hardly or not at all)
Integrated KT approach	The KT plan begins at the knowledge production stage and takes into account the needs and context of the knowledge users throughout the project	The KT plan begins after the knowledge has been produced but takes into account the needs and the context of the knowledge users	The KT plan begins after the knowledge has been produced and does not take into account the needs and context of the knowledge users
End-of-grant approach	The approach fosters ongoing interaction between researchers and users	The approach fosters occasional interaction between researchers and users	The approach hardly fosters or does not at all foster interaction between researchers and users
Dimension 7: KT evaluation			
Criterion	A (Predominantly)	B (Moderately)	C (Hardly or not at all)
Evaluation of the KT process	The plan calls for ongoing evaluation of the KT process and adjustments during implementation	The plan calls for a few evaluation procedures (such as indicators) but the approach is not yet defined	The plan does not include any evaluation of the KT process
Dimension 8: Resources			
Criterion	A (Predominantly)	B (Moderately)	C (Hardly or not at all)
Feasibility (availability of human, physical and financial resources)	Provision has been made for the necessary resources (funding, staff, material, time) to carry out the plan	Provision has been made for resources to carry out the plan but they are deemed insufficient (e.g. their lack is identified as an obstacle in the context analysis)	The resources required to carry out the plan are unavailable or not specified in the plan
